# The Importance of Bulk Viscoelastic Properties in “Self-Healing” of Acrylate-Based Copolymer Materials

**DOI:** 10.1021/acsmacrolett.3c00626

**Published:** 2023-12-11

**Authors:** Yuqi Zhao, Hanshu Wu, Rongguan Yin, Krzysztof Matyjaszewski, Michael R. Bockstaller

**Affiliations:** † Department of Materials Science & Engineering, Carnegie Mellon University, 5000 Forbes Avenue, Pittsburgh, Pennsylvania 15213, United States; ‡ Department of Chemistry, Carnegie Mellon University, 4400 Fifth Avenue, Pittsburgh, Pennsylvania 15213, United States

## Abstract

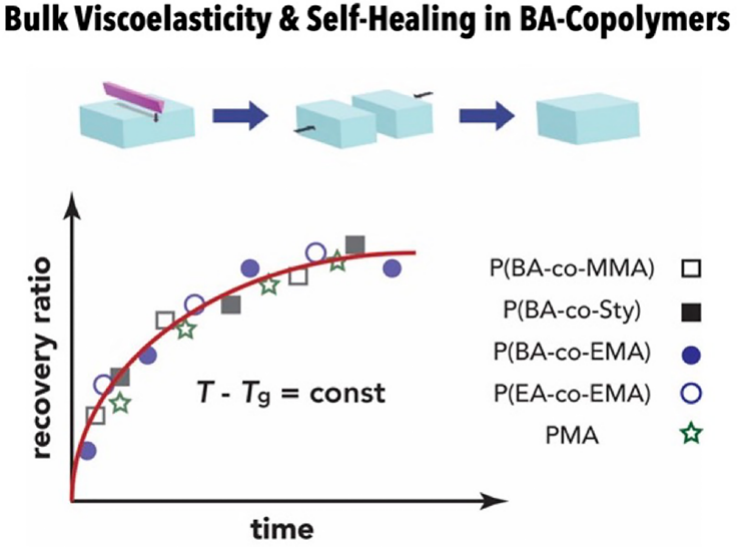

“Self-healing” has emerged as a concept to increase the functional stability and durability of polymer materials in applications and thus to benefit the sustainability of polymer-based technologies. Recently, van der Waals (vdW)-driven “self-healing” of sequence-controlled acrylate-based copolymers due to “key-and-lock”- or “ring-and-lock”-type interactions has generated considerable interest as a viable route toward engineering polymers with “self-healing” ability. This contribution systematically evaluates the time, temperature, and composition dependence of the mechanical recovery of acrylate-based copolymer and homopolymer systems subject to cut-and-adhere testing. “Self-healing” in *n*-butyl acrylate/methyl methacrylate (BA/MMA)- or *n*-butyl acrylate/styrene (BA/Sty)-based copolymers with varying composition and sequence is found to correlate with the bulk viscoelastic properties of materials and to follow a similar trend as other tested acrylate-based homo- and copolymers. This suggests that “self-healing” in this class of materials is more related to the chain dynamics of bulk materials rather than composition- or sequence-dependent specific interactions.

Endowing materials with “self-healing” capability, i.e., the ability to recover functionality after incurring structural damage, has attracted much interest in the past decade due to the growing relevance of increasing longevity and hence sustainability of polymeric products and devices.^1−7^ The recovery from structural damage is commonly accomplished at elevated temperatures, when chain dynamics is fast enough to enable welding and interdiffusion across interfaces. However, fast chain dynamics requires high temperatures or a low cohesive energy density (and thus low elastic modulus) of materials. A ubiquitous challenge for realizing “self-healing” polymers has thus been to enable structure recovery on practical time scales and temperatures in materials with high enough cohesive energy density (CED) to realize engineering-relevant moduli. To afford rapid “self-healing” in materials with high modulus (and hence slow dynamics), two strategies have been developed that aim to decouple the dynamical properties in the bulk and damage regions. Extrinsic “self-healing” involves the dispersion of low molecular healing agents through vascularization or encapsulation.^8−10^ In contrast, intrinsic “self-healing” is accomplished by dynamic covalent bonds as in Diels–Alder^11^ and disulfide chemistries,^12^ or through supramolecular noncovalent chemistries such as hydrogen bonding^13−16^ or metal ion coordination.^17^ Damage results in localized breaking of the dynamic bond linkages. This increases the dynamics of chains in the damage region and promotes the reconstitution of bonds during “healing” of the fracture surface.^18,19^ It is important to note that the criteria for considering a recovery process as “self-healing” are subjective and generally determined based on application needs and practicality rather than mechanistic arguments. This introduces an inherent ambiguity and process dependence of a material’s classification as “self-healing” and motivates the use of quotation marks in the remainder of this paper.

Recently, an intriguing approach to realize van der Waals-based dynamic-bond-type interactions was proposed in which the deliberate placing of pendant groups in copolymers gives rise to a microstructure with increased CED via “key-and-lock” or “ring-and-lock” interactions.^20−25^ In 2018, Urban and co-workers observed that copolymers based on *n*-butyl acrylate (BA) and methyl methacrylate (MMA) with about equimolar composition (50–55 mol % BA), synthesized via atom transfer radical polymerization (ATRP), featured self-healing.^21^ Assuming a random sequence of copolymers, the authors proposed “key-and-lock” interactions between alternating BA/MMA repeats to raise the CED and promote “self-heal ability”. Ouchi et al. successfully achieved BA/MMA copolymers with alternating sequence and reported a faster recovery rate and increased CED (viz. higher glass transition temperature and elastic modulus) than the corresponding statistical sequence copolymers.^26^ However, the statistical copolymer was synthesized by free radical polymerization, which results in nonuniform mixtures (i.e., a set of gradient copolymer chains with varying composition but overall equimolar BA:MMA stoichiometry) rather than a homogeneous statistical sequence copolymer. Thus, the results cannot be seen as unequivocal evidence of a “key-and-lock” microstructure in BA/MMA copolymers. In independent studies, our group explored the “self-healing” and thermomechanical properties of BA/MMA copolymers in which microstructure was precisely controlled by means of atom transfer radical polymerization (ATRP).^27−31^ Systematic investigations of BA/MMA copolymers with varying molar composition (encompassing the compositional range reported in the original work) and controlled statistical (*stat*), gradient (*grad*), and alternating (*alt*) sequence confirmed a faster rate of recovery for alternating and statistical sequence copolymer as compared to the gradient analogues. However, glass transition temperatures (*T*_g_) were not affected by the copolymer sequence; also the Young’s modulus and toughness were found to be reduced in *alt*/*stat* copolymers as compared to the gradient analogues. Interestingly, the width of the glass transition temperature of copolymers with equimolar stoichiometry was found to increase from the *alt*-to-*stat*-to-*grad* sequence structure. Small angle neutron scattering (SANS) analysis revealed the formation of MMA-clusters within gradient copolymers, thus confirming a heterogeneous microstructure. Our analysis thus attributed the accelerated “self-healing” kinetics of *alt* and *stat* copolymers to the faster dynamics of polymer chains since a more uniform microstructure avoids pinning of chains within high *T*_g_ cluster regions, thus promoting chain interdiffusion.^27,29^ While our results did not preclude the existence of a “key-and-lock”-type mechanism, they did raise questions about the relative relevance of chain dynamics and bulk viscoelasticity versus the impact of specific interactions due to the sequence structure of BA/MMA copolymers on the “self-healing” process.

To further elucidate the role of bulk viscoelasticity on the “self-healing” of BA/MMA copolymers, this contribution presents a systematic evaluation of the effect of copolymer chemical composition on structure and property recovery in acrylate-based copolymers. A series of copolymers composed of various acrylate and methacrylate combinations, sequence structures, and compositions were synthesized by activators regenerated by electron transfer (ARGET)-ATRP (Figure 1a). Statistical and gradient sequences with both equimolar and asymmetric compositions were evaluated to gauge the impact of “key-and-lock” arrangements (via variation of the frequency of suitable dyads) on the recovery process. All (co)polymer systems featured a comparable glass transition temperature and, hence, chain dynamics. Poly(methyl acrylate) (PMA) homopolymer with a *T*_g_ value similar to P(BA-*co*-MMA) was included in the analysis as a reference for which no specific interactions are expected nor have been noted in the literature. To evaluate the ability of materials to “self-heal”, cut-and-adhere followed by tensile testing using equivalent conditions to prior studies was employed. The results demonstrate that all (co)polymer systems with similar *T*_g_ values as compared to equimolar P(BA-*stat*-MMA) displayed comparable structure and property recovery rates. Likewise, BA/MMA statistical copolymers with asymmetric composition (and thus a substantially reduced frequency of BA/MMA alternating dyads) featured “self-healing” at temperatures near the glass transition. The results demonstrate that bulk viscoelastic properties (in conjunction with a corroborative definition of the desirable kinetic attributes of “self-healing”) are sufficient to rationalize “self-healing” in symmetric BA/MMA copolymers. While trends in the glass transition temperature of the various copolymer systems suggest that specific interactions could indeed exist, these are concluded to play a secondary role in the restoration of the structure and properties of damaged BA/MMA copolymer materials.

**Figure 1:**
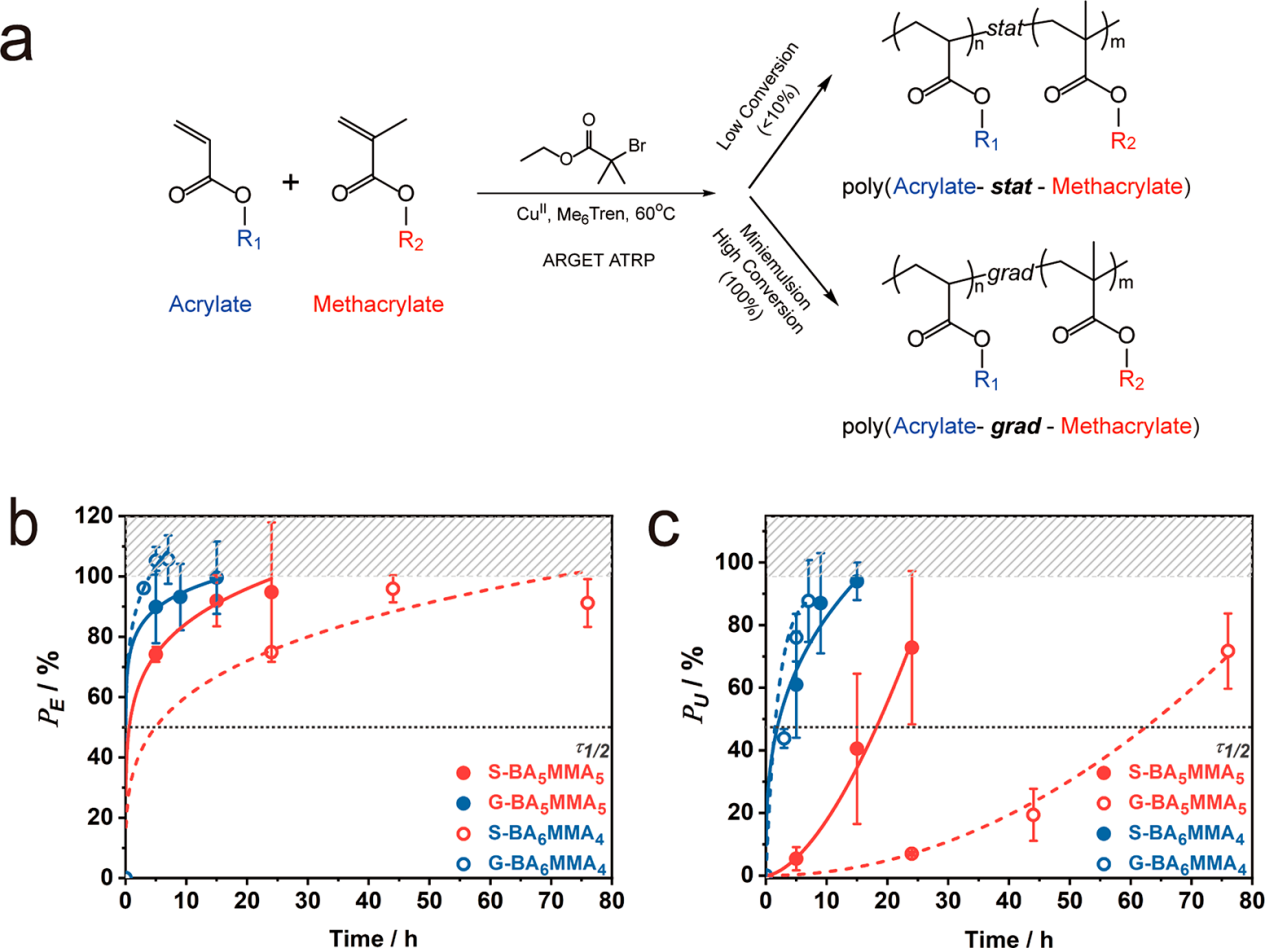
(a) Illustration of synthesis routes for poly(acrylate-*stat*-methacrylate) and poly(acrylate-*grad*-methacrylate). (b and c) Property recovery rates for equimolar copolymers S-BA_5_MMA_5_ and G-BA_5_MMA_5_ after rejoining of films at room temperature (21 °C) for Young’s modulus (*P*_E_, b) and toughness (*P*_U_, c). Values in panels b and c are normalized with respect to pristine film properties. Lines are introduced to guide the eye.

The following abbreviations are used to identify the respective copolymer systems *X*-R1_*Y*_R2_*Z*_ with *X* = S/G representing *stat* and *grad* sequence structures; R1 and R2 representing the respective composition of repeats, i.e., butyl acrylate (BA), ethyl acrylate (EA), ethyl methacrylate (EMA), methyl methacrylate (MMA), and styrene (Sty); and *Y* and *Z* representing the respective molar fraction of repeats. Three S-BA_*Y*_MMA_*Z*_ and three G-BA_*Y*_MMA_*Z*_ with similar molecular weight and dispersity but different BA compositions (40, 50, and 60 mol %, respectively) were synthesized following the methods that were reported recently (Figure 1a and Figure S1).^28,30,31^ To identify the role of composition-specific interactions in the structure reconstitution process, a set of statistical copolymers consisting of BA/Sty, BA/EMA, and EA/EMA were synthesized. Compositions were chosen such that the respective glass transition temperatures approximately matched the value of the equimolar BA/MMA system for which “self-healing” was reported. A homopolymer, poly(methyl acrylate) (PMA), with similar *T*_g_ was used as a reference to evaluate the role of the copolymer nature of materials. To allow comparison of the results, all polymers featured a similar molecular weight close to the materials in the original work on “key-and-lock enabled self-healing”.^21^Table 1 summarizes the molecular characteristics of all polymer systems that were the subject of the present study along with their glass transition and recovery characteristics determined by differential scanning calorimetry (DSC) and dynamic mechanical analysis (DMA) of “healed” systems. In agreement with previous reports on BA/MMA copolymers,^31^ the glass transition temperatures of both S and G sequence copolymers increased with MMA composition (Figure S2b). Statistical copolymers featured near identical *T*_g_’s compared to gradient analogues of similar composition but displayed more narrow glass transitions. This confirmed prior reports on BA/MMA copolymers with varied sequence structures that attributed the broader glass transition to increased heterogeneity in gradient systems.

**Table 1:** Characteristics of Copolymer and P(Methyl Acrylate) Homopolymer Systems

Entry^a^	*x*_R1_ (mol %)^b^	*x*_R2_ (mol %)^b^	*M*_n_^c^	*M*_w_/*M*_n_^c^	Conversion (%)^c^	*T*_g_^d^ (°C)	*T*_g_ Range (°C)^d^	τ_1/2_ R.T. (h)^e^
S-BA_4_MMA_6_	40.4	59.6	53,690	1.37	8.9	23	[5, 37]	NA
S-BA_5_MMA_5_	51.3	48.7	55,980	1.37	9.6	5	[−10, 15]	18.4
S-BA_6_MMA_4_	59.2	40.8	58,640	1.53	8.8	–2	[−18, 9]	2.3
G-BA_4_MMA_6_	38.6	61.4	54,170	1.32	100	28	[−4, 55]	NA
G-BA_5_MMA_5_	50.7	49.3	57,460	1.31	100	9	[−11, 39]	64.3
G-BA_6_MMA_4_	59.6	40.4	53,690	1.37	100	–9	[−28, 27]	1.7
								
S-EA_4_EMA_6_	41.4	58.9	44,270	1.16	12.5	11	[−5, 19]	NA
S-BA_3_EMA_7_	28.8	71.2	34,680	1.20	11.4	10	[−10, 21]	NA
S-BA_5_Sty_5_	52.1	47.9	37,750	1.15	11.9	11	[−6, 21]	NA
PMA	100	0	24,500	1.07	35.0	17	[2, 24]	NA

a Reaction conditions were listed in the Supporting Information.

b Determined by ^1^H NMR.

c Determined by SEC.

d Determined by DSC.

e Self-healing half-time of fracture toughness calculated from strain–stress curves measured by DMA. Methacrylate was replaced by styrene for S-BA_5_Sty_5_.

To evaluate self-healing capability, films with thicknesses of 0.2 mm were subjected to cut-and-adhere processing (detailed procedures are provided in the Supporting Information). The elastic (Young’s) modulus and fracture toughness were determined after defined annealing periods, and the half-time of recovery τ_1/2_ (representing the time to recover one-half of the initial mechanical performance of the pristine material) was introduced as a quantitative measure for the “self-heal” efficacy. Two properties were evaluated, i.e., Young’s modulus *E* and fracture toughness *U*, that were measured by tensile testing using a constant strain rate of 0.05 s^–1^. Figure 1b and c depict the evolution of recovery ratios *P*_E_ and *P*_U_ (defined as the elastic modulus and fracture toughness normalized by the respective value of the pristine material, i.e., *P*_E_ = *E*(*t*)/*E*_0_ and *P*_U_ = *U*(*t*)/*U*_0_) for equimolar BA/MMA copolymers, i.e., compositions within the expected compositional range for “key-and-lock” interactions (BA:MMA = 0.5–0.55).^21^ In agreement with prior findings, the statistical sequence copolymer displayed a faster recovery of Young’s modulus and toughness as compared to the gradient analogue. This was due to the increased heterogeneity of gradient copolymers that resulted in the pinning of chains, thus delaying the reconstitution of the material microstructure.^27,32−35^ However, Figure 1b and c also reveal that “self-healing” was not unique to equimolar compositions. Both S-BA_6_MMA_4_ and G-BA_6_MMA_4_ featured recovery of fracture toughness after 2.3 and 1.7 h, i.e., 700% and 3700% faster as the symmetric analogues at 21 °C (S-BA_5_MMA_5_, 18.4 h; G-BA_5_MMA_5_, 64.3 h). This result underlines the role of diffusive processes on structure reconstitution, which are accelerated in the softer S/G-BA_6_MMA_4_ systems. While structure reconstitution at *T* > *T*_g_ (i.e., in the quasi-liquid state) might be considered trivial, the result does illustrate the challenge associated with the rather qualitative definition of “self-healing materials” as predominantly solid materials (i.e., the storage modulus exceeds the respective loss modulus) that feature some defined level of structure and property reconstitution within some predefined time range and temperature (such as 24 h at 294 K).

To evaluate the role of bulk viscoelasticity vs specific interactions on the “healing” process, we re-evaluated the recovery rates for S-BA_5_MMA_5_ and G-BA_5_MMA_5_ at 30 °C, i.e., at *T* – *T*_g_ = 25 °C, a comparable difference to S/G-BA_6_MMA_4_ tested at 21 °C. We note that a more accurate approach to evaluate the effect of chain dynamics would be based on the Williams–Landel–Ferry equation; however, this approach was not found to be practical here.^33,36^Figure 2 reveals that S-BA_5_MMA_5_ features a 300% acceleration of recovery at 30 °C. In particular, the half time for recovery of fracture toughness decreased to τ_1/2_ = 4.6 h (Figure 2b), comparable to that of the S/G-BA_6_MMA_4_ system. Even more pronounced acceleration of recovery was observed for the gradient analogue G-BA_5_MMA_5_ that resumed 50% of the initial fracture toughness after 3.6 h (Figure 2d).

**Figure 2:**
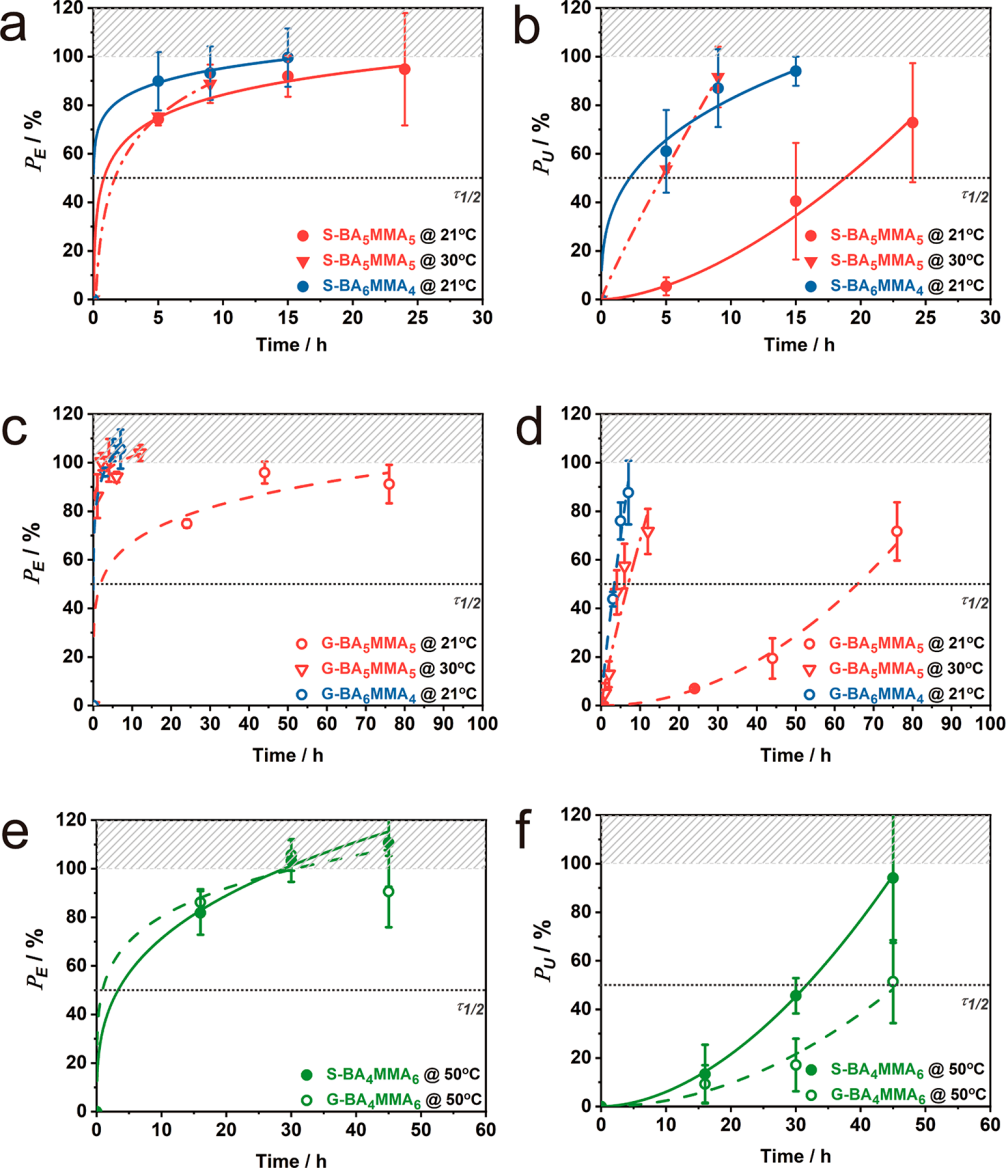
Property recovery after rejoining of films at room temperature (21 °C) and 30 °C for S-BA_5_MMA_5_ and S-BA_6_MMA_4_: (a) Young’s modulus (*P*_E_) and (b) toughness (*P*_U_), as well as G-BA_5_MMA_5_ and G-BA_6_MMA_4_: (c) Young’s modulus (*P*_E_) and (d) toughness (*P*_U_). Property recovery after rejoining of films at 50 °C for S-BA_4_MMA_6_ and G-BA_4_MMA_6_: (e) Young’s modulus (*P*_E_), (f) toughness (*P*_U_). Values in parts a–f are normalized with respect to pristine film properties. Lines are introduced to guide the eye.

Further confirmation of the role of chain dynamics was provided by analysis of the MMA-rich BA_4_MMA_6_ copolymers. Property recovery tests were performed at 50 °C to maintain a similar distance to the glass transition, i.e., *T* – *T*_g_ = 25 °C. Interestingly, both systems displayed property recovery as seen in Figure 2e and f with half times of recovery of Young’s modulus of about 3 h and toughness just outside the defined 24 h range (30 h for S-BA_4_MMA_6_ and 45 h for G-BA_4_MMA_6_, respectively). To further evaluate the role of chain dynamics on “self-healing”, the effect of temperature on both macroscopic and microscopic relaxation was determined by creep and dynamic mechanical analysis (Figure S5). Creep analysis of copolymers upon application of a constant stress (10 kPa) in the linear regime (Figure S5a) revealed an increase of nonrecoverable strain (i.e., flow) for S/G-BA_5_MMA_5_ copolymers at 30 °C. The temperature dependence of chain relaxation times (determined from tan(δ) measurements, Figure S5c and d) was similar to the observed changes in healing times. For example, the half time of toughness recovery of S-BA_5_MMA_5_ decreased from 18.4 h at 21 °C to τ_1/2_ = 4.6 h at 30 °C. This is comparable to the shift of relaxation times from 2π/ω_0_ = 3.3 s at 20 °C to 0.16 s at 30 °C, where ω_0_ is the frequency corresponding to the maximum of tan(δ). Furthermore, at both temperatures, G-BA_5_MMA_5_ displayed slower relaxation as the statistical analogue, in agreement with the correspondingly slower recovery times.

While the results above highlight the role of dynamical processes and bulk viscoelasticity on the “self-healing” of BA/MMA copolymers, they do not provide conclusive insight into the presence of structure-related specific interactions. To discern the presence of specific interactions that have been proposed for approximately equimolar BA/MMA copolymers (and, more recently, BA/Sty statistical copolymers^24^), a series of copolymers and homopolymers with comparable *T*_g_ to BA_5_MMA_5_ were synthesized.

Poly(ethyl acrylate-*stat*-ethyl methacrylate) (S-EA_4_EMA_6_), poly(butyl acrylate-*stat*-ethyl methacrylate) (S-BA_3_EMA_7_), and poly(butyl acrylate-*stat*-styrene) (S-BA_5_Sty_5_) copolymers were synthesized using ARGET ATRP. Since the stoichiometry of copolymers was chosen such as to approximately match the *T*_g_ of BA_5_MMA_5_, copolymers with a varying degree of stoichiometric asymmetry (depending on the *T*_g_ of the respective homopolymers) were synthesized. Thus, based on molecular constitution *and* composition, specific interactions such as “key-and-lock” or “ring-and-lock” that require a high frequency of suitable dyads were not expected in these systems. All materials were subjected to cut-and-adhere testing under equivalent conditions. Figure 3 summarizes the property recovery that was measured after varying the testing time. The figure reveals that all three copolymers featured complete mechanical property recovery within 6 h at 21 °C (Figure 3b). This confirmed that specific interactions of the type “key-and-lock” or “ring-and-lock” were not required to initiate structure and property recovery but that “self-healing” is rather the result of favorable dynamical characteristics of the copolymer systems under test conditions. To further rule out effects relating to a particular copolymer structure, poly(methyl acrylate) (PMA) with *T*_g_ ∼ 17 °C was synthesized and evaluated using cut-and-adhere testing. Complete recovery of mechanical properties was achieved within 6 h of “self-healing”. Since an atactic homopolymer such as PMA is not known to form “interlocked” structures in the solid state, this result provides further support of the hypothesis that self-healing in copolymer materials is predominantly related to bulk viscoelasticity rather than specific interactions such as key-/ring-and-lock.

**Figure 3:**
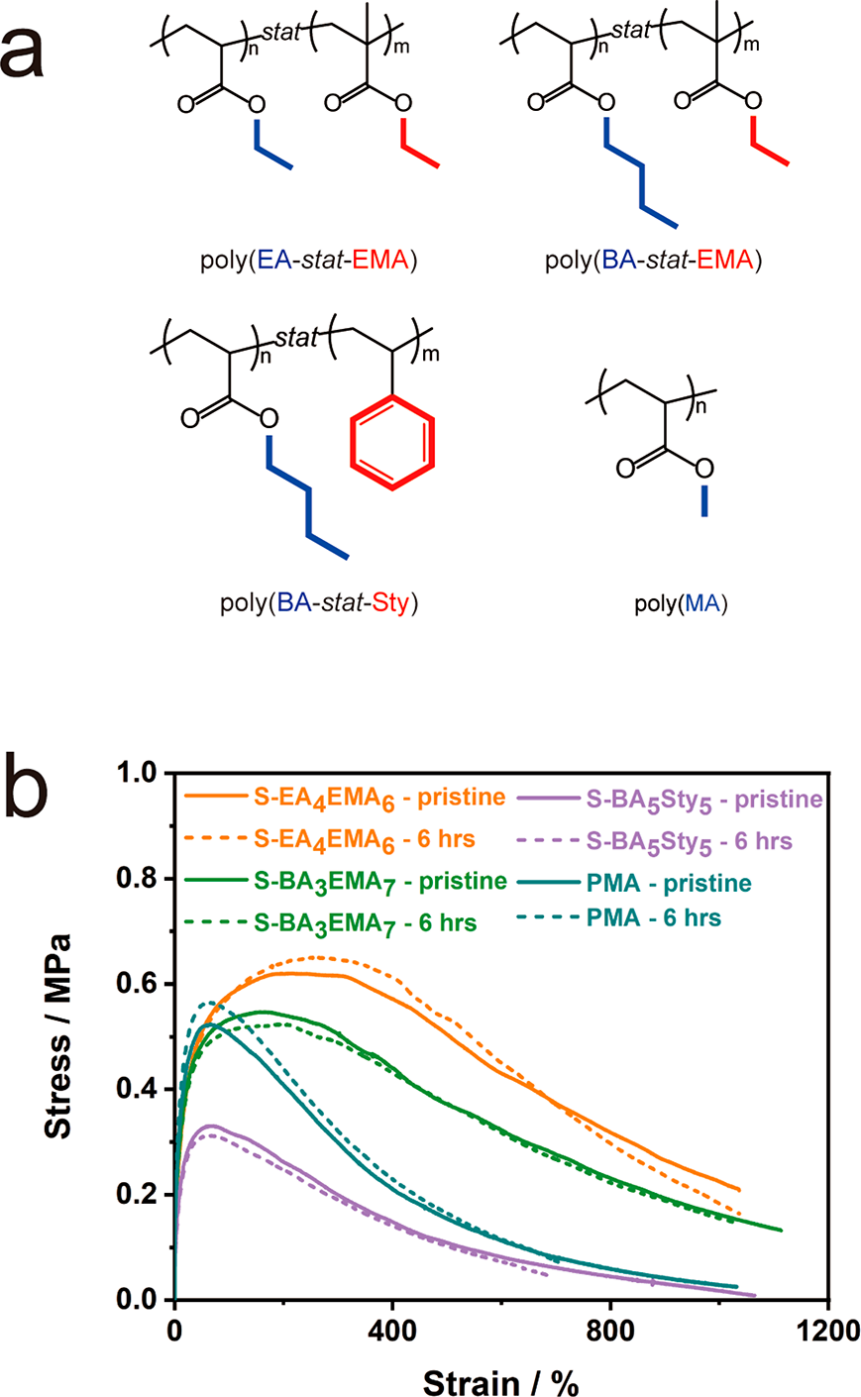
(a) Chemical formulas for P(EA-*stat*-EMA), P(BA-*stat*-EMA), P(BA-*stat*-Sty), and PMA. (b) Strain–stress curves for pristine and 6 h healing cut-and-healed films at room temperature (21 °C).

We note that these results do not disprove the existence of “key-and-lock”-type interactions in BA/MMA copolymer systems. To illustrate this point, Figure 4 depicts the discrepancy between the experimental and predicted glass transition temperatures of copolymer systems. Predictions were made based on the Fox equation and the *T*_g_’s of the respective homopolymers (with comparable molecular weight) that were synthesized using ATRP. Interestingly, the figure reveals that the *T*_g_’s of both BA/MMA and BA/Sty copolymers exceeded the predicted estimates while other copolymer systems featured *T*_g_’s below the calculated values. The overshoot of the predicted values is largest at about symmetric composition, which could be indicative of a promoting effect of BA/MMA dyads on the cohesive energy density. However, the width of the glass transition (indicated by range-bars in Figure 4) which is intrinsically broader for *stat* (and *grad*) copolymer systems renders this conclusion ambiguous.

**Figure 4:**
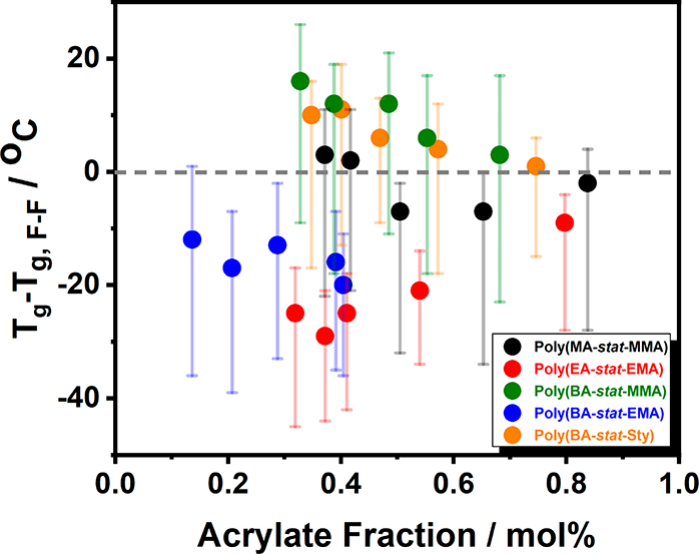
Temperature difference between copolymer *T*_g_ and its corresponding Fox-predicted glass transition temperature, i.e., *T*_g_ – *T*_g,F_. Range bars indicate the width of the glass transition.

Also, the Fox equation, while being widely used for its practicality, makes several idealizing assumptions such as random mixing between monomer components, equal changes of the heat capacity of the components at the glass transition as well as negligible volume of mixing across the entire compositional range which limit its predictive power in the present case.^37^

In conclusion, the systematic evaluation of the time, temperature, and composition dependence of the mechanical recovery of acrylate-based copolymer and homopolymer systems subjected to cut-and-adhere testing reveals the importance of bulk viscoelastic properties for “self-healing”. The similar “healing” characteristics even of nonstochiometric copolymer compositions under conditions of comparable chain dynamics suggest that specific interactions (such as “key-and-lock” or “ring-and-lock”) that have been proposed for BA/MMA or BA/Sty random copolymers do not dominate structure or property recovery in these systems. Further research is needed to better understand the parameters governing “key-and-lock”-type interactions and the conditions under which these interactions can drive “self-healing” processes. Future study, for example, using vibrational spectroscopy on copolymers with varied or controlled tacticity, could clarify the role of specific interactions in BA-based copolymers. Although the present study indicates the importance of bulk viscoelastic properties for “self-healing”, sequence-controlled copolymers present an intriguing platform for the design of polymer materials with enhanced recovery behavior. This is because of the wider range of control parameters (such as sequence and composition) as compared to homopolymers, which provides opportunities to decouple microscopic dynamics from structural stability. Thus, these materials hold the prospect to realize materials combining “high modulus” with “self-healing” ability. This, however, will be the subject of a forthcoming publication.

## Supporting Information Available

Details of synthesis procedures, characterization procedures (DSC, tensile test, DMA, creep test, self-healing test), heat flow and derivative heat flow curves, strain–stress, self-healing, dynamic mechanical, and creep data (PDF)
