# Supplementary material for: The Importance of Bulk Viscoelastic Properties in “Self-Healing” of Acrylate-Based Copolymer Materials
Source: ACS Macro Lett. 2023 Dec 11;13(1):1–7. doi: 10.1021/acsmacrolett.3c00626 (PMC10795469; doi:10.1021/acsmacrolett.3c00626)
Supplement: Supplementary file 1 — mz3c00626_si_001.pdf [file mz3c00626_si_001.pdf]

Supporting Information

The Importance of Bulk Viscoelastic Properties in ‘Self-Healing’ of Acrylate-Based Copolymer Materials

Yuqi Zhao[†], *Hanshu Wu*[‡], *Rongguan Yin*[‡], *Krzysztof Matyjaszewski*[*, ‡], *Michael R. Bockstaller*[*, †]

[†]Department of Materials Science & Engineering, Carnegie Mellon University, 4400 Fifth Avenue, Pittsburgh, Pennsylvania 15213, USA

[‡] Department of Chemistry, Carnegie Mellon University, 5000 Forbes Avenue, Pittsburgh, Pennsylvania 15213, USA

Experimental Section

Procedures.

Procedures for the synthesis of linear PBA-statistical-PMMA copolymers via ARGET ATRP.

Initiator (EBiB), monomer: BA and MMA, solvents (anisole), $CuBr_2$, and Me_6TREN, molar ratios shown in supporting information, were mixed thoroughly in a sealed Schlenk flask, followed by degassing by bubbling with nitrogen. Then the $Sn(EH)_2$ was injected into the Schlenk flask to activate the catalyst complex, and the flask was immediately put into an oil bath set at the desired temperature. The conversion was monitored and controlled under 10% by ^{1}H-NMR. The final products were precipitated in cold methanol and then dissolved and stored in THF.

Molar ratios for the synthesis of S-BA4MMA6, S-BA5MMA5, S-BA6MMA4, G-BA4MMA6, G-BA5MMA5, and G-BA6MMA4 via ARGET ATRP.

For S-BA4MMA6, Initiator (EBiB, 0.004 mL), monomer: MMA (5.64 g, 6.0 mL) and BA (11.84 g, 13.3 mL), solvents (anisole 10 mL), $CuBr_2$ (0.005 g in 1 mL DMF), Me_6TREN (0.01 mL), and $Sn(EH)_2$ (0.025 g, 0.02mL). For S-BA5MMA5, Initiator (EBiB, 0.005 mL), monomer: MMA (3.95g, 4.2 mL) and BA (12.01 g, 13.5 mL), solvents (anisole 10 mL), $CuBr_2$ (0.005 g in 1 mL DMF), Me_6TREN (0.01 mL), and $Sn(EH)_2$ (0.025 g, 0.02mL).For S-BA6MMA4, Initiator (EBiB, 0.004 mL), monomer: MMA (3.76g, 4.0 mL) and BA (7.57 g, 8.5 mL), solvents (anisole 10 mL), $CuBr_2$ (0.005 g in 1 mL DMF), Me_6TREN (0.01 mL), and $Sn(EH)_2$ (0.025 g, 0.02mL).

Procedures for the synthesis of linear PBA-gradient-PMMA copolymers via ARGET ATRP.

Initiator (EBiB, 0.018 mL), monomer (BA, 2.24 g, 2.5 mL), (MMA, 1.75 g, 1.86 mL), and HD (0.4 g), were mixed thoroughly to form the oil phase. CuIIBr2/TPMA stock solution (0.05 M in 18.2 MΩ·cm ultrapure water, 0.57 mL), NaBr (0.24 g, 0.1 M), and SDS (0.25 g, 6.2 wt% to comonomers) were dissolved in 17.73 mL of ultrapure water. The oil and aqueous solutions were

mixed (total volume ≈ 23.56 mL), placed in an ice bath, and homogenized by an ultrasonic probe sonicator, amplitude = 25 % for 1 min (application and rest time of 1 s each, 2 min in total). The mixture was degassed by bubbling with nitrogen. A stock solution of AsAc in ultrapure water (0.05 g/mL) was prepared. Then, slowly injected by syringe pump (at 0.05 mL/h) into the Schlenk flask to activate the catalyst complex and the flask was immediately put into an oil bath set at the desired temperature. The conversion and molecular weight (MW) of the polymer were monitored by 1H-NMR and SEC, respectively. The final linear copolymers were soluble and stored in THF.

Procedures for the synthesis of linear PEA-statistical-PEMA/ PBA-statistical-PEMA/ PBA-statistical-PS / PMA-statistical-PMMA/ PBA-statistical-PMMA copolymers via ARGET ATRP.

Initiator (EBiB, 0.005 mL), monomers, solvents (anisole 5 mL), $CuBr_2$ (0.005 g in 1 mL DMF) and Me_6TREN (0.01 mL), were mixed thoroughly in a sealed Schlenk flask. The amount of monomers utilized for the synthesis of each copolymer is listed in Table S1. The mixture was degassed by bubbling with nitrogen. A stock solution of $Sn(EH)_2$ in anisole was prepared. The reaction solution was degassed by nitrogen purging, then the $Sn(EH)_2$ solution was injected into the Schlenk flask to activate the catalyst complex, and the flask was immediately put into an oil bath set at the desired temperature. The conversion was monitored and controlled under 10% by ^1H-NMR, and molecular weight (MW) of the polymer was monitored by SEC. The linear copolymers were soluble and stored in THF.

Table S1. Monomers and monomer feeding ratios for synthesis of each copolymer.

**Entry**	**monomer 1 (amount)**	**monomer 2 (amount)**
S-EA_{80}-EMA_{20}	EA (9mL/ 8.46g)	EMA (0.5mL/ 0.46g)
S-EA_{54}-EMA_{46}	EA (8mL/ 7.52g)	EMA (1.5mL / 1.37g)
S-EA_{37}-EMA_{63}	EA (6.5mL/ 6.11g)	EMA (2mL/ 1.82g)
S-EA_{41}-EMA_{59}	EA (7mL/ 6.58g)	EMA (2.5mL /2.28g)
S-EA_{32}-EMA_{68}	EA (6mL /5.64g)	EMA (3.5mL/ 3.19g)
S-BA_{40}-EMA_{60}	BA (8mL/ 7.12g)	EMA (2mL/ 1.83g)
S-BA_{39}-EMA_{61}	BA (7mL/ 6.23g)	EMA (2.5mL/ 2.28g)
S-BA_{29}-EMA_{71}	BA (6mL/ 5.34g)	EMA (3.5mL/ 3.19g)
S-BA_{21}-EMA_{79}	BA (5mL/ 4.45g)	EMA (4.5mL/ 4.11g)
S-BA_{14}-EMA_{86}	BA (4mL/ 3.56g)	EMA (5.5mL/ 5.02g)
S-BA_{75}-Sty_{25}	BA (9.5mL/ 8.46g)	Sty (0.5mL/ 0.45g)
S-BA_{57}-Sty_{43}	BA (8.5mL/ 4.90g)	Sty (1.5mL/ 1.36g)
S-BA_{47}-Sty_{53}	BA (7.5mL/ 6.68g)	Sty (2.5mL/ 2.28g)
S-BA_{40}-Sty_{60}	BA (6.5mL/ 5.79g)	Sty (3.5mL/ 3.18g)
S-BA_{35}-Sty_{65}	BA (5.5mL/ 4.90g)	Sty (4.5mL/ 4.10g)
S-MA_{84}-MMA_{16}	MA (9mL/ 8.55g)	MMA (0.5mL/ 0.47g)
S-MA_{65}-MMA_{35}	MA (8mL/ 7.60g)	MMA (1.5mL/ 1.41g)
S-MA_{50}-MMA_{50}	MA (7mL/ 6.65g)	MMA (2.5mL/ 2.35g)
S-MA_{42}-MMA_{58}	MA (6mL/ 5.70g)	MMA (3.5mL/ 3.29g)

S-MA_{37}-MMA_{63}	MA (5mL/ 4.75g)	MMA (4.5mL/ 4.23g)
S-BA_{68}-MMA_{32}	BA (11.5mL/ 10.24g)	MMA (1.5mL/ 1.41g)
S-BA_{55}-MMA_{45}	BA (10.5mL/ 9.35g)	MMA (2.5mL/ 2.35g)
S-BA_{48}-MMA_{52}	BA (13.5 mL/ 12.02 g)	MMA (4.0 mL/ 3.76 g)
S-BA_{39}-MMA_{61}	BA (8.5mL/ 7.57g)	MMA (4.0 mL/ 3.76g)
S-BA_{33}-MMA_{67}	BA (7mL/ 6.23g)	MMA (4.5mL/ 4.23g)

Procedures for synthesis of linear PMA homopolymer via ARGET ATRP.

Initiator (EBiB, 0.01 mL), monomer: MA (4.75 g, 5 mL), solvents (anisole 5 mL), CuBr 2 (0.005 g in 1 mL DMF) and Me 6 TREN (0.01 mL), were mixed thoroughly in a sealed Schlenk flask. The mixture was degassed by bubbling with nitrogen. A stock solution of Sn(EH) 2 in anisole was prepared. The reaction solution was degassed by nitrogen purging, then the Sn(EH) 2 solution was injected into the Schlenk flask to activate the catalyst complex, and the flask was immediately put into an oil bath set at the desired temperature. The conversion was monitored and controlled under 10% by 1 H-NMR, and molecular weight (MW) of the polymer was monitored by SEC. The linear copolymers were soluble and stored in THF.

Procedures for fabrication of a bulk film.

Linear copolymers were dispersed in THF *via* sonication. After the solution was stirred for 24 h, the bulk dispersions were transferred into 15 mm × 5 mm rectangular Teflon molds. The solvent was slowly evaporated over 48 h at room temperature, generating transparent nanocomposite films with a thickness of 0.1-0.2 mm. The residual solvent was removed from the bulk films by transferring them to a vacuum oven and slowly increasing the temperature at the rate of 10 °C per

24 h to 120 °C. At least five specific bulk films of the same composition were investigated to systematically study the thermo-mechanical properties of the nanocomposite films.

Characterization.

Nuclear Magnetic Resonance Spectroscopy (NMR). Conversion of polymerization was monitored by ^{1}H NMR on a Bruker Advance 500 MHz NMR instrument in $CDCl_3$ at room temperature.

Size Exclusion Chromatography (SEC). Number-average molecular weights (M_n) and molecular weight distributions (MWD) of samples were determined by size exclusion chromatography (SEC). The SEC was conducted with an Agilent 1260 Iso pump and Waters 410 differential refractometer using PSS columns (Styragel 10^5, 10^3, 10^2 Å) with THF as an eluent at 35 ºC and at a flow rate of 1 mL min^{-1}. Linear PMMA standards were used for calibration. Diphenylethylene and toluene were used as internal standards for the system.

Differential Scanning Calorimetry (DSC). The glass transition temperature (T_g) of linear copolymers were measured by differential scanning calorimetry (DSC) with TA Instrument QA-2000. The same procedure was run three times, each involving the following steps: (1) Equilibrate at 25.00 °C, (2) Isothermal for 1.00 min, (3) Ramp 20.00 °C/min to -90.00 °C, (4) Isothermal for 1.00 min, (5) Ramp 20.00 °C/min to 160.00 °C, (6) Isothermal for 1.00 min, (7) Ramp 20.00 °C/min to -90.00 °C, (8) Isothermal for 1.00 min, (9) Ramp 20.00 °C/min to 160.00 °C, (10) Isothermal for 1.00 min, (11) Ramp 20.00 °C/min to -90.00 °C, (12) Isothermal for 1.00 min, (13) Ramp 20.00 °C/min to 160.00 °C, (14) Isothermal for 1.00 min, (15) Jump to 25.00 °C. The DSC data were analyzed with a TA Universal Analysis instrument, and T_g was directly acquired.

Dynamic Mechanical Analysis (DMA).

Tensile test: the linear copolymer bulk films are tested in the tensile mode by using DMA (TA RSA-G2). The film thickness was between 100-200 μm. The samples were stretched at a constant tensile rate of 0.05 mm/mm/s at room temperature.

Damping property measurement: The damping property was measured through dynamic mechanical analysis (DMA, TA RSA-G2) in a frequency range of 0.1-100 Hz at room temperature, with application of 0.1% strain. All the samples were tested at least three times for consistency.

Creep test: Creep experiments were performed on pristine specimens with applied stress of 10 kPa for 90 seconds at room temperature (TA RSA-G2), followed by a recovery time of 180 seconds in which stress was removed.

Cut-and-Adhere Testing.

Self-healing of bisected bulk films: a bulk film (dimension: 15mm x 5mm x 150 mm) was severed rapidly by a sharp razor blade to get clean-cut surfaces. Then, the two parts were gently rejoined, and two cutting surfaces were softly reattached without any buckling to prevent edges overlap within 1 min and allowed to self-heal for a specific time under ambient conditions. After that time, the same film after healing was characterized by a tensile test as described above. Then, it was compared to the pristine samples and calculated the recovery ratio.

Supporting data:

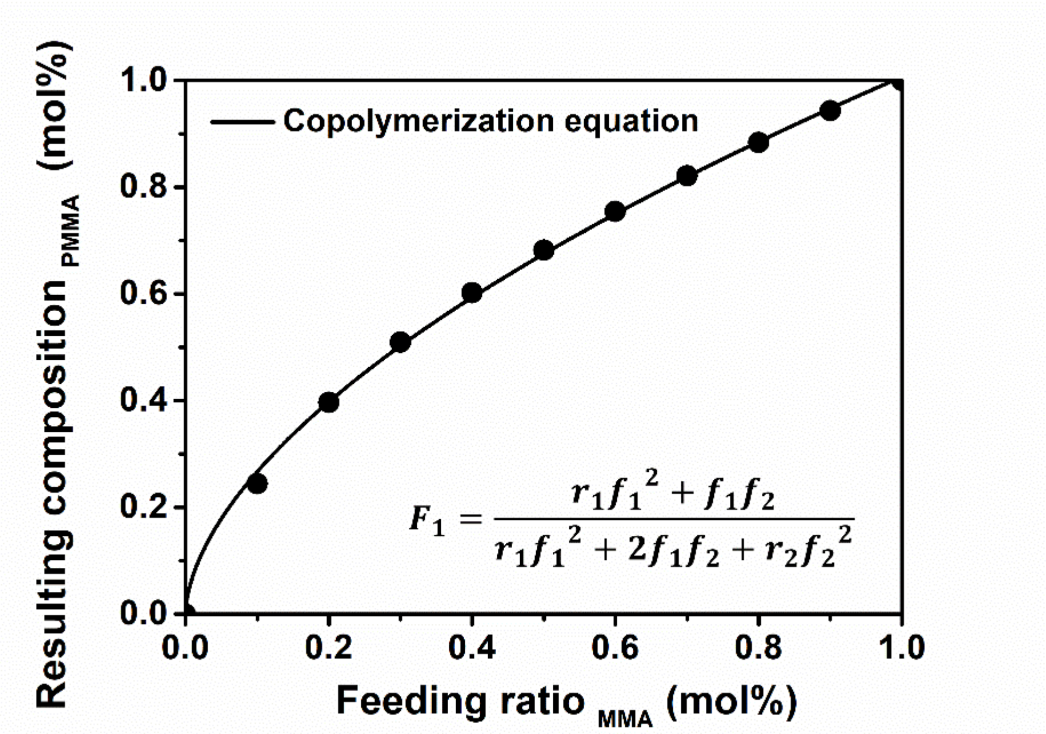

Figure S1. Statistical samples prepared at low conversion to avoid comonomer feed drift and the corresponding initial feed ratios (xMMA mol%) were used to prepare random copolymers with the resulting compositions (xPMMA mol%) according to the copolymerization equation.

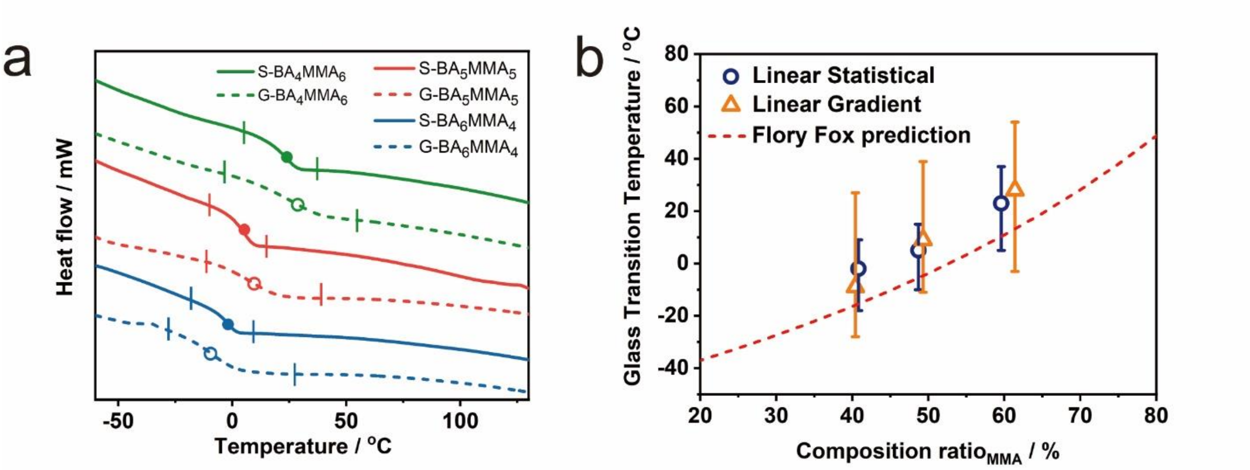

Figure S2. (a) DSC heat curves. The Tgs are highlighted with solid points in the figure. (b) The Tgs and Flory Fox prediction. All curves were recorded during the 3rd heating/cooling run at a heating rate of 20 ºC/min.

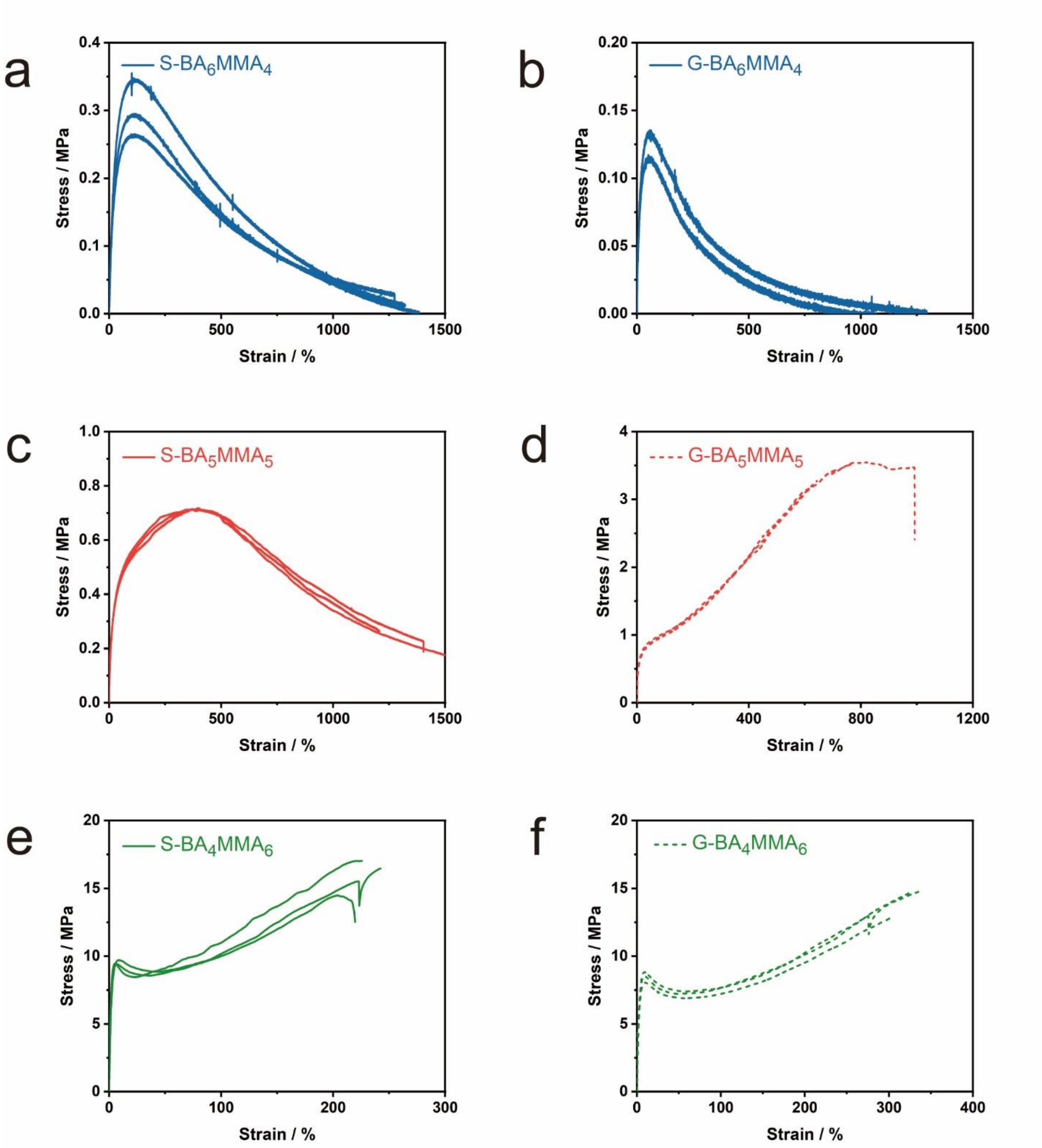

Figure S3. Strain-stress curves: (a) S-BA6MMA4, (b) G-BA6MMA4, (c) S-BA5MMA5, (d) G-BA5MMA5, (e) S-BA4MMA6, (f) G-BA4MMA6, . All samples were measured three times with different bulk films as shown in same color lines in the figures.

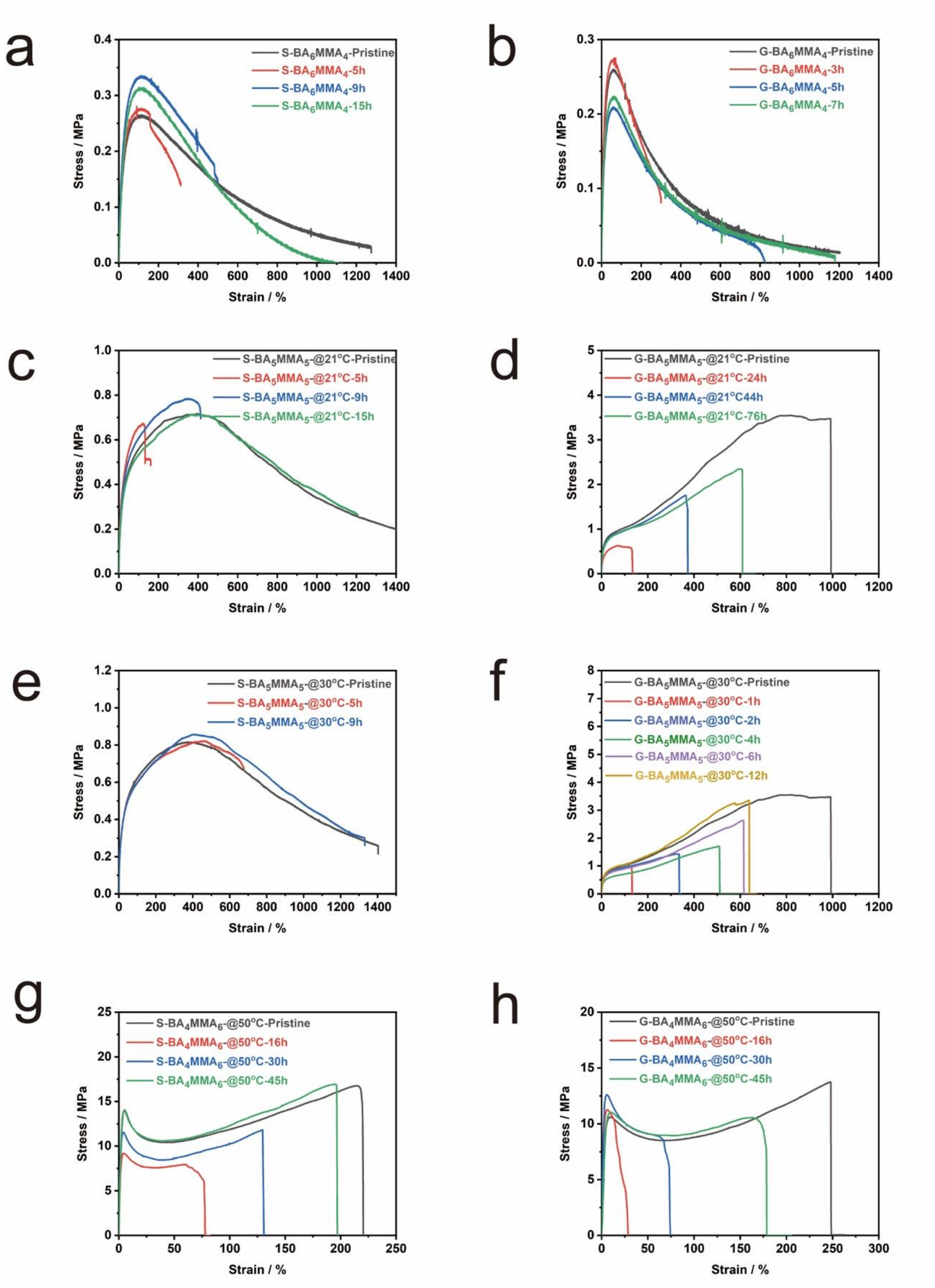

Figure S4. Strain-stress curves for pristine and damaged-and-healed films: (a) S-BA6MMA4, (b) G-BA6MMA4, (c) S-BA5MMA5, (d) G-BA5MMA5, (e) S-BA5MMA5 at 30 ^oC, (f) G-BA5MMA5 at 30 ^oC, (g) S-BA4MMA6 at 50 ^oC, (h) G-BA4MMA6 at 50 ^oC.

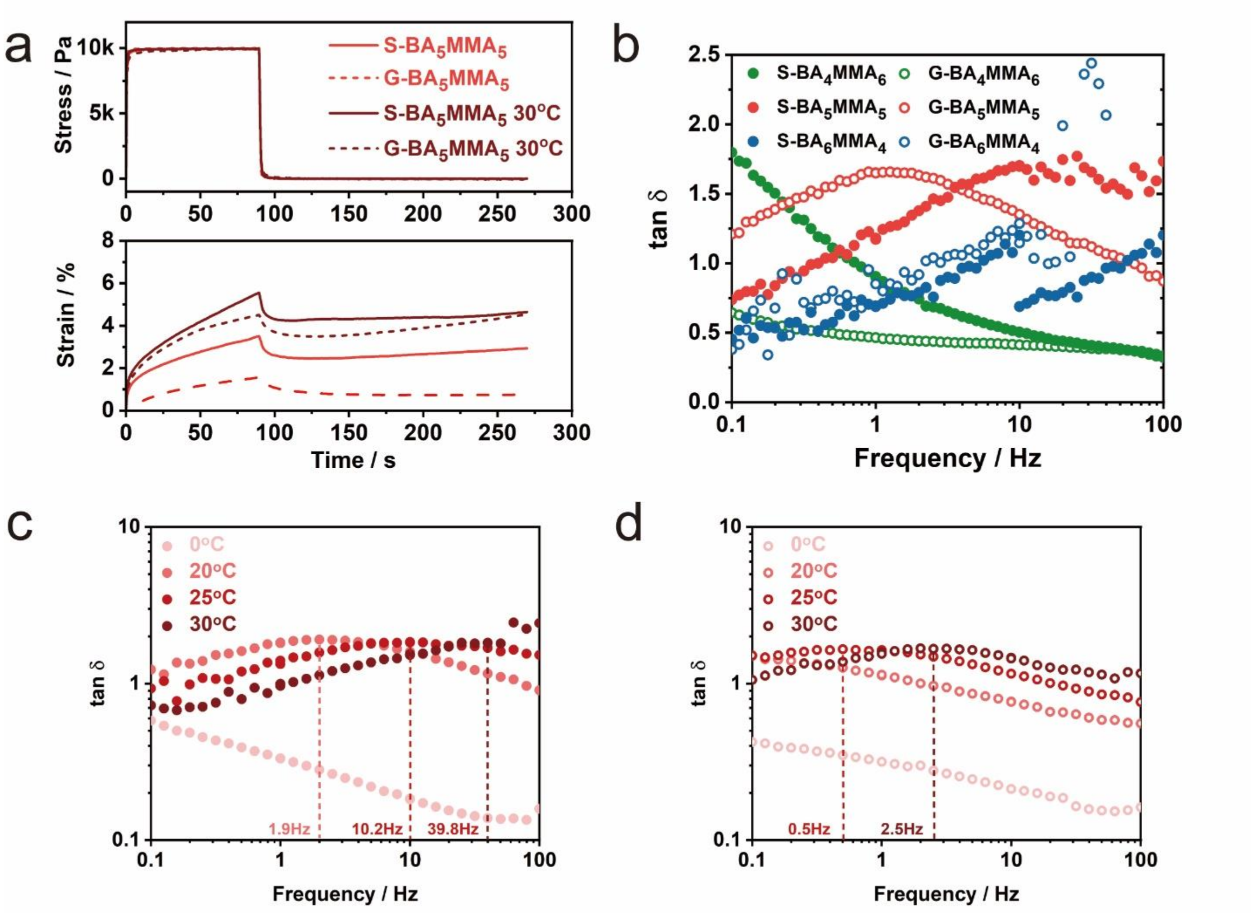

Figure S5. (a) Creep behaviors for S/G-BA5MMA5 at R.T. (21ºC) and 30ºC; (b) dynamic mechanical analysis damping properties for S/G; (c) tan d at different temperature for S-BA5MMA5; (d) tan d at different temperature for G-BA5MMA5

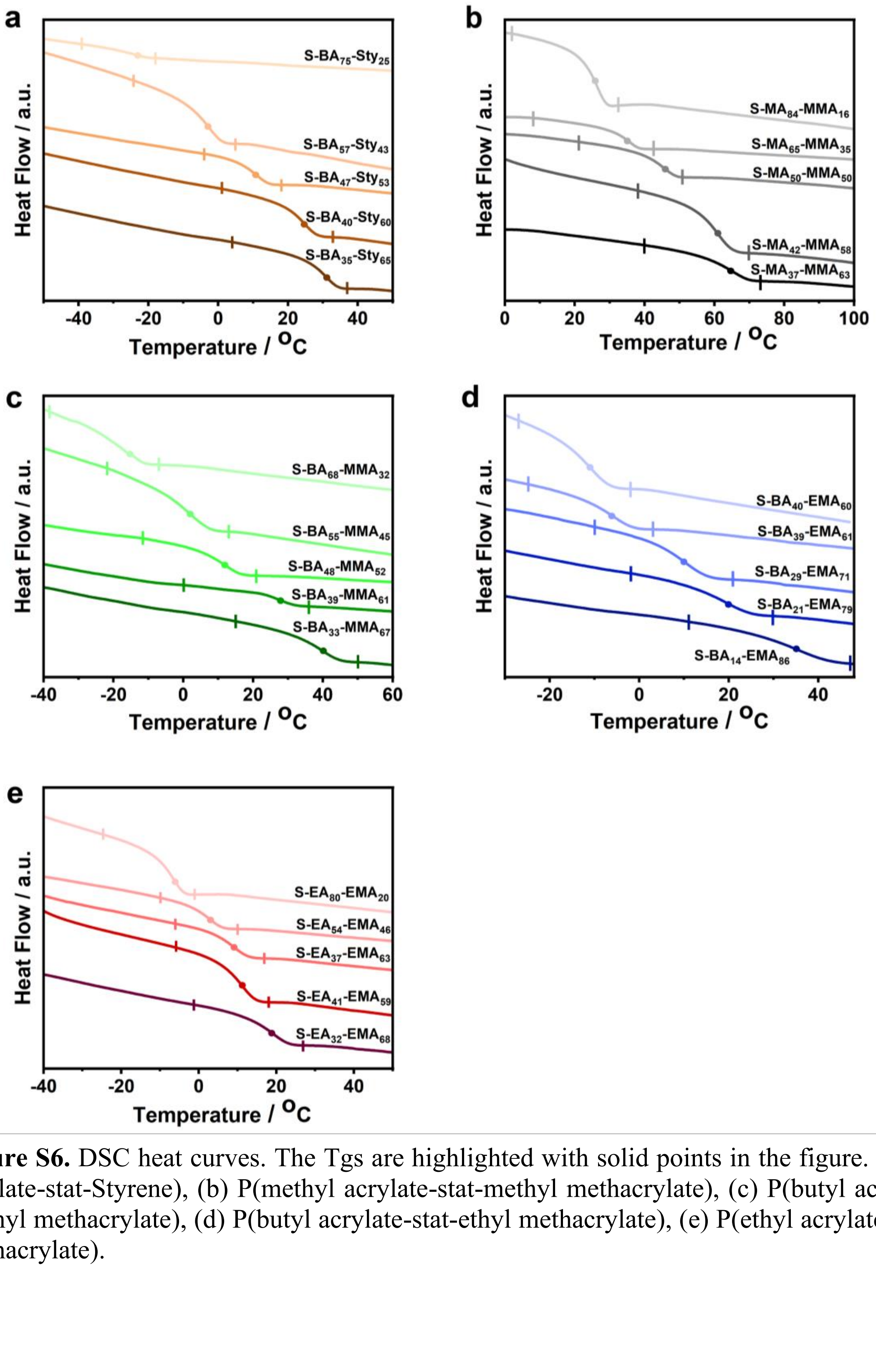

Figure S6. DSC heat curves. The Tgs are highlighted with solid points in the figure. (a) P(butyl acrylate-stat-Styrene), (b) P(methyl acrylate-stat-methyl methacrylate), (c) P(butyl acrylate-stat-methyl methacrylate), (d) P(butyl acrylate-stat-ethyl methacrylate), (e) P(ethyl acrylate-stat-ethyl methacrylate).

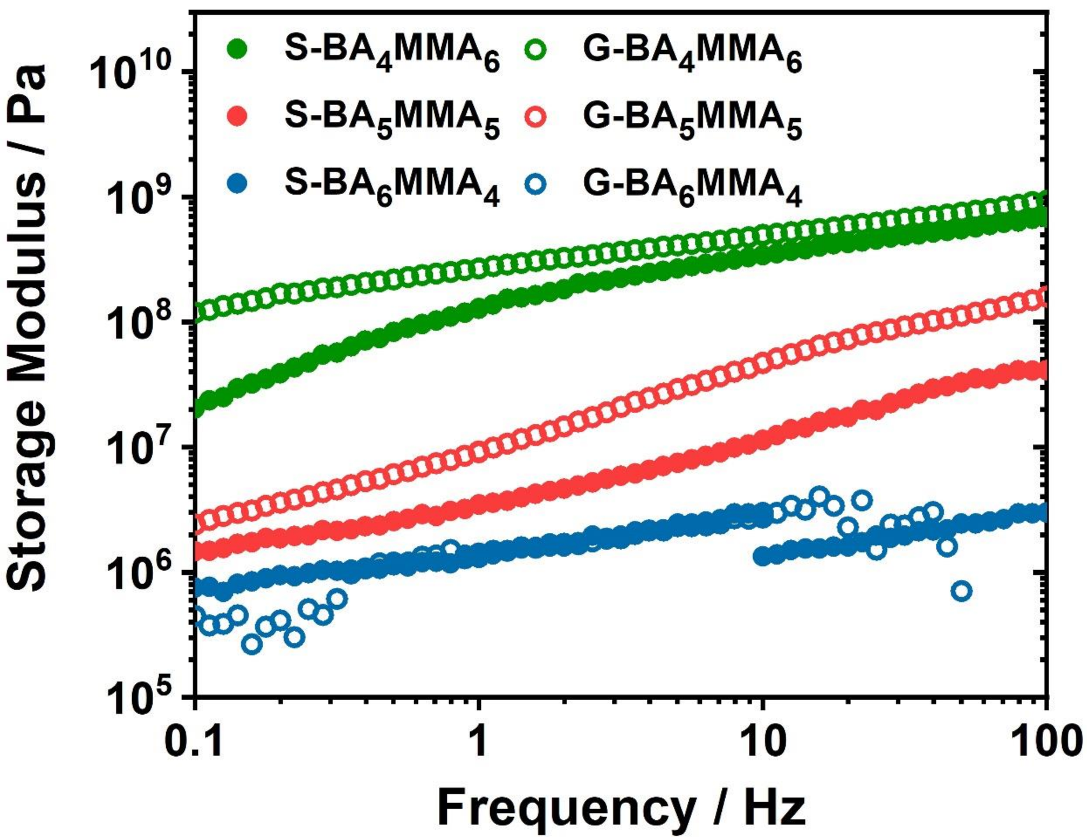

Figure S7. Dynamic mechanical analysis storage modulus for S/G.
